# Acceptability of a Digital Early Years Language Support Service for Caregivers of Children Who Have SLCN and/or Are Multilingual

**DOI:** 10.1111/1460-6984.70210

**Published:** 2026-03-01

**Authors:** Emily Hancock, Colin Bannard, Silke Fricke, Penny Levickis, Rachel McGurk Isaacs, Cristina McKean, Julian Pine, Gideon Salter, Rosie Sherlock, Kiera Solaiman, Danielle Matthews

**Affiliations:** ^1^ School of Psychology University of Sheffield Sheffield UK; ^2^ Department of Linguistics and English Language University of Manchester Manchester UK; ^3^ School of Allied Health Professions, Nursing and Midwifery University of Sheffield Sheffield UK; ^4^ REEaCh Centre, Faculty of Education University of Melbourne Melbourne Australia; ^5^ Sheffield Methods Institute School of Education University of Sheffield Sheffield UK; ^6^ Department of Education University of Oxford Oxford UK; ^7^ Department of Psychology University of Liverpool Liverpool UK; ^8^ School of Psychology Liverpool Hope University Liverpool UK

**Keywords:** English as an additional language (EAL), infancy, language intervention, smartphone, Special Education Needs (SEN), telehealth

## Abstract

**Background:**

Parent‐child interaction (PCI) interventions have the potential to mitigate early‐identified risks of Speech, Language and Communication Needs (SLCN). PCI interventions can be delivered at Universal, Targeted and Specialist levels, but evidence for effectiveness at the Universal level is lacking, especially for some populations. We examine the acceptability of a universal PCI intervention for two underserved groups: children who have SLCN and/or are multilingual. For the former group we also explore acceptability of a supplementary, targeted intervention.

**Aim:**

This study aimed to: (a) evaluate the acceptability of a digital early years PCI support service—comprising a universal text‐message service delivering BBC Tiny Happy People videos and targeted speech and language therapy following the Early Language Identification Measure & Intervention (ELIM‐I); (b) establish the interest of families with children who have, or are at risk of, SLCN (*N* = 61) and/or are multilingual (*N* = 26) in utilising the service, and explore their perceptions regarding merits and drawbacks of the service, and elicit recommendations for improvements.

**Methods & Procedures:**

We employed a mixed‐methods approach. Quantitative data were collected via questionnaires based on the Theoretical Framework of Acceptability. Qualitative data were gathered through semi‐structured online interviews. Families of children with SLCN provided prospective acceptability data after reviewing three videos, indicating their view of receiving similar weekly video content via text message. Those who then opted to try the text service for a month provided retrospective acceptability data, with additional questions for participants who received the targeted online ELIM‐I intervention. Multilingual families received the service for three months before providing retrospective acceptability data.

**Outcomes & Results:**

Quantitative analyses revealed that all acceptability ratings were high on average, though there was individual variability. Reflexive thematic analysis of caregivers’ qualitative data identified three central themes: (a) demand for trustworthy guidance to address uncertainty; (b) positives including service suitability for busy family life, personalisation, human connection and reassurance, enjoyment and perceived efficacy; (c) a need for inclusive content, especially for children with complex SLCN.

**Conclusions & Implications:**

There is a clear desire for early digital services to help caregivers support their children's language development. Acceptability was generally high. Caregivers wanted to see their family represented in video content. This was largely successful for the multilingual group with content celebrating home languages. Caregivers of children with SLCN sometimes felt under‐represented and recommended demonstrating support strategies appropriate for their child's age and stage of development.

**WHAT THIS PAPER ADDS:**

*What is already known on this subject*

- PCI interventions have the potential to mitigate the risk of SLCN from the early years. There has been interest in creating digital interventions that can reach families universally at low cost and BBC Tiny Happy People have developed video content along these lines. However, previous evaluations of digital services using BBC Tiny Happy People materials, have excluded multilingual families and families who have children with/at risk of SLCN.

*What this paper adds to existing knowledge*

- This study highlights that a digital support service comprising universal weekly text messages sharing BBC Tiny Happy People video content and targeted online ELIM‐I intervention is acceptable and desirable to families whose children have SLCN and/or are multilingual. Caregivers highlighted necessary changes to cater for diversity, particularly the need for appropriate content for families whose children have SLCN.

*What are the potential or actual clinical implications of this study?*

- Findings support the use of a public health framework to provide families with accessible and evidence‐based support (in this case, a universal digital service to promote PCI in the early years with an additional, targeted ELIM‐I intervention for those with SLCN). This could remove pressure from in‐person services by preventing need, supporting triaging, enabling families to support their child whilst waiting for speech and language therapy and to be more ready for specialist intervention if this is needed. Selecting content that best matches families’ characteristics increases acceptability.

**Practitioner Points:**

1. Digital Services are Acceptable as an Initial Layer of Intervention

Caregivers rated the digital language support service highly. They felt that it addressed their curiosity and concern about their child's development and that its benefits persisted regardless of whether caregivers were accessing other support. This indicates that a digital, universal service can function as an effective initial layer of intervention, providing support to individuals on waiting lists and contributing to the management of speech and language therapy caseloads.

2. Inclusive and Diverse Content is Essential to Avoid Caregiver Distress and Harm

Content that is representative of family diversity is essential in universal digital language support services. Caregiver feedback highlighted the profound impact of representation: a multilingual family articulated feeling ‘connected with the people’ when presented with relevant content. Conversely, a perceived lack of content reflective of complex needs was associated with participant distress and reduced engagement or withdrawal from the service. These experiences highlight an ethical responsibility to develop inclusive and representative content that reflects a wide range of developmental trajectories, family structures and cultures.

3. The Speech and Language Therapist's Pivotal Role in Personalising Universal Digital Services

The study demonstrated that the human element of the service, such as personalised messages and text message and phone or video call interaction, was highly valued by caregivers. This human connection helped offset the limitations of a universal digital service, providing the opportunity to clarify misunderstandings and a sense of being listened to and understood. This finding indicates that a successful digital service is not about replacing human interaction; it is about using digital platforms to complement and extend the reach of their professional role.

## 1 Introduction

Many children arrive at primary school without the language skills that help them to thrive (Norbury et al. 2016). These Speech, Language and Communication Needs (SLCN) can occur in isolation or alongside other Special Educational Needs (SEN). Public health approaches can address this with a preventative framework, whereby a universal service is offered to all families and additional, targeted support is offered to those at higher risk of SLCN (McKean and Reilly 2023b; Molloy et al. 2021). This ‘stacked intervention’ approach stands to yield cumulative benefits for children with SLCN. However, its effectiveness depends upon the universal component successfully reaching and being acceptable for families with SLCN (Skivington et al. 2021). Moreover, since universal approaches aim to promote equity (Carey et al. 2015), in the UK's multilingual society materials also need to be acceptable to families using a range of languages at home.

A key approach to mitigate risk is to support caregivers to promote their child's development through responsive interactions (Burgoyne et al. 2018; McGillion et al. 2017). While evidence for the effectiveness of early‐years Parent‐Child Interaction (PCI) interventions is mixed (Burgoyne et al. 2024; Heidlage et al. 2020), interest in them has increased due to concerns about increased SLCN following the COVID‐19 pandemic (Zuniga‐Montanez et al. 2025). Interest in exploring digital interventions has also increased given the higher ownership of smartphones in families, practitioner adoption of technological solutions and the financial and workforce constraints on speech and language therapy services (Ansari et al. 2022; Nitsche et al. 2021; Royal College of Speech and Language Therapists 2025). However, the few studies that have evaluated digital early years PCI support services (e.g., Matthews et al. 2023) tend to have focused on socio‐economic disadvantage as a risk factor and excluded families with children who have SLCN and/or are multilingual.

Community‐based universal initiatives aimed at enhancing PCI have the potential to foster early language development, both to those families and children who may need a small boost and those requiring more intensive support (Beecher and Van Pay 2021). However, there is limited evidence supporting interventions implemented at the universal level (Levickis et al. 2022; Roberts et al. 2019). While there has been significant research on parent‐mediated intervention to support children with SLCN (Tosh et al. 2016) including specifically for children with SLCN experiencing economic disadvantage (Gibbard et al. 2024), we know of no qualitative work exploring the acceptability of a universal intervention for children with or at risk of SLCN (but see Dunstan et al. 2024; Pila et al. 2019). This is problematic if universal services are to confer a cumulative benefit for this population. Moreover, while there has been a call for more research on SLCN to consider the needs of multilingual families (Nair et al. 2022), we still need to better understand how universal services delivered in the majority community language are received by families speaking minority languages at home, whether or not their child has SLCN. Finally, while online interventions have had some success for caregivers whose children have SLCN (e.g., Tang et al. 2024), we currently do not know how acceptable a universal online service would be for caregivers whose children have or are at risk of SLCN. Exploring this is important given that such families might be highly motivated to use and able to access a digital universal service but the generic content might need to be adapted to their needs.

In the UK, the BBC recently developed a new digital service for parents and caregivers that includes a large bank of short videos about how and why to support early child language through interaction (BBC Tiny Happy People, part of BBC parenting: www.bbc.co.uk/tiny‐happy‐people). A universal digital intervention was developed by Matthews et al. (2023) to deliver these videos to caregivers via weekly texts that contain links to the BBC Tiny Happy People videos. In an initial evaluation (Matthews et al. 2023), this text‐message service was well received with 91% of families reporting that they would recommend it to other caregivers. Furthermore, it was found to promote caregiver linguistic responsiveness and infant communication though it did not improve vocabulary outcomes (Matthews et al. 2023; Salter et al. 2025). While the evaluation excluded those with children who had SLCN diagnosed at baseline, feedback from caregivers whose children later went on to have an SLCN diagnosed was that it would be helpful to combine the universal text message service with online support from a Speech and Language Therapist (SLT) where appropriate. We therefore developed a stacked digital service with a universal component (text messaging) working in combination with a digital adaptation of the existing ELIM‐I intervention (McKean et al. 2022; Newcastle University 2025). While there is evidence to suggest the ELIM‐I is acceptable to caregivers (when delivered in person: McKean et al. 2023a; McKean et al. in preparation) there is a need to explore how a complex digital intervention with a universal component is received by caregivers whose children have SLCN.

Data from health visitor checks in England for 2023–2024 show that 13% of children aged 24 to 30 months were below the expected level in communication skills, a rise from 11% in 2018 (Department for Health and Social Care 2025). Families with a child who has or is at risk of SLCN (due to a medical or developmental condition associated with language learning difficulties) might not find a universal service acceptable or relevant. Moreover, since some families may already be accessing support services, it is important to explore whether additional digital support would be a help or hindrance.

According to the Department for Education (2025), in England 30% of children in nurseries reported English as an additional language. Multilingual families raising children with English as an additional language may also have unique needs and preferences. Multilingualism is not a risk factor for language difficulties in itself (Peña et al. 2023) but it is unclear how caregivers would receive a universal service primarily featuring monolingual, English‐speaking families albeit with extra content celebrating multilingualism. Moreover, it is likely that, in multilingual families with a child with SLCN, the challenges faced are different to those of monolingual households. It is therefore important to understand how this combination of factors influences the acceptability of the service.

Thus, the aim of the current study was to:

- Investigate the acceptability of a universal digital service to support PCI in the early years with an additional, targeted intervention.
- Understand whether caregivers of children who had SLCN and/or were multilingual would show interest in this type of service, and if so, which service elements would be valued and what are the recommendations to improve acceptability.

## 2 Materials and Methods

### 2.1 Research Design and Setting

**FIGURE 1:**
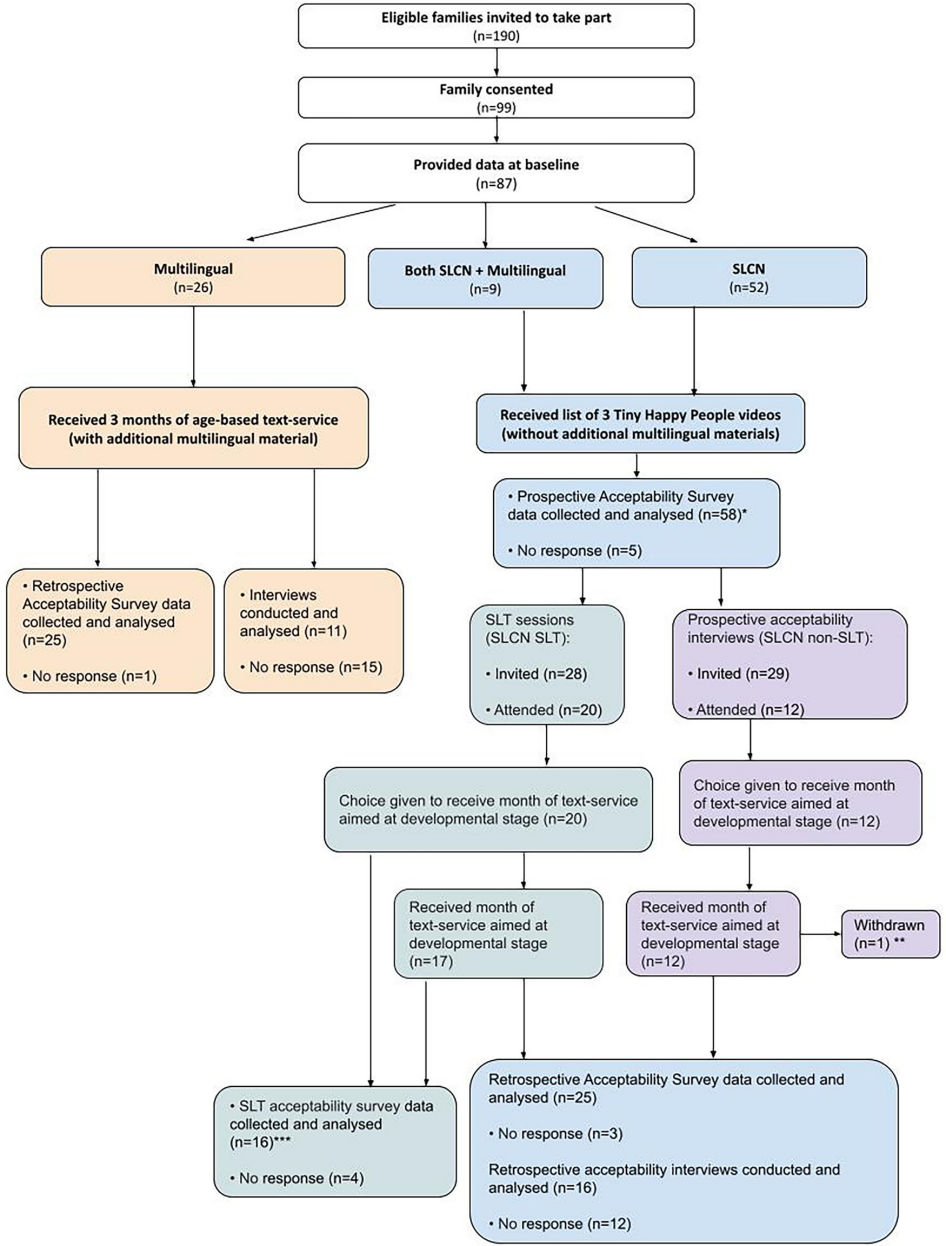
Flow Chart of study participation. *Note*: *Late submissions from two participants who did not proceed to the next stage. ** One participant withdrew midway, finding the service unacceptable and preferring their existing SLT support. ***Completed by the attending caregiver (in one case, not the primary correspondent).

We used a mixed methods approach to explore acceptability to caregivers with questionnaires yielding acceptability ratings on the seven dimensions of the Theoretical Framework of Acceptability (Sekhon et al. 2022) and interviews exploring caregivers’ views in more depth (see Figure 1). Supplementary materials can be found on the OSF at: https://osf.io/ty9d7/. Ethical approval was received from the School of Psychology Ethics Sub‐committee at the University of Sheffield in October 2024 (058667). All participants provided written, informed consent. Standards for Reporting Qualitative Research guidelines are followed (O'Brien et al. 2014, Checklist: Supplementary Information A).

### 2.2 Participant Recruitment

Participants were invited to take part in this study if they were ineligible for a randomised controlled trial (RCT) that was running in parallel (with nation‐wide recruitment through marketing companies and social media). Both the present study and the RCT had the following inclusion criteria:

1. Child born between February 2023 and November 2023 (Group 1) or August 2021 and May 2022 (Group 2). For this study, both age groups are combined.
2. Home postcode of the primary caregiver within deciles 1–5 of the Office of National Statistics Index of Multiple Deprivation for England or country equivalent for Wales, Scotland and Northern Ireland.
3. Caregiver had access to an internet‐enabled device to receive text links and watch videos.

Additionally, the RCT had the following exclusion criteria (which this study did not automatically apply as explained below):

4. Birth weight below 2.5 kg (5lbs 8oz)
5. Child born before full term (37+ weeks)
6. Child had a developmental or medical condition known to affect language development or a diagnosed/suspected language delay
7. Child heard English less than 50% of the time at home

For the present study, all invited families met inclusion criteria (1–3), but we invited those excluded from the RCT due to criteria 4–7, as follows:

***SLCN group***: Those either with a developmental or medical condition known to affect language development (Supplementary Information B: Full list of conditions) or a diagnosed/suspected language delay were included, even if the infant had a birth weight below 2.5 kg (5lbs 8oz) and/or was born before full term (37+ weeks. Hee et al. 2020). Multilingual children included.
***Multilingual group***: Those who heard English less than 50% of the time at home (Supplementary Information B: Full list of languages) and were not in the SLCN group. All families were competent in English as recruitment, video content and study participation were in English only.

At baseline all caregivers completed a survey comprising demographic questions (see Table 1), the Ages and Stages Questionnaire Communication and Gross Motor subscales (Squires and Bricker 2009) and, as relevant, health and home language questions (see Supplementary Information B for more detail). Participants received a £10 Love2Shop voucher for each study component completed.

**TABLE 1:** Participant demographics.

Demographic feature		Participant characteristics			
**Caregiver relationship to child** **(*N* = 88** ^*^)		‘Mum’ *n* = 86	‘Dad’ *n* = 2	Other *n* = 0	
		(98%)	(2%)	(0%)	

**Caregiver education**		No degree qualification	Degree qualification		
		*n* = 31	*n* = 57		
		(35%)	(65%)		

**IMD (*N* = 87** ^*^)	IMD 1	IMD 2	IMD 3	IMD 4	IMD 5
	*n* = 17	*n* = 19	*n* = 18	*n* = 16	*n* = 17
	(19%)	(22%)	(20%)	(19%)	(19%)

**Child sex** **(*N* = 88** ^*^)		Male	Female		
		*n* = 51	*n* = 37		
		(58%)	(42%)		

**Child ethnicity**	White	Asian	Mixed ethnicity	Black	
	*n* = 62	*n* = 13	*n* = 8	*n* = 5	
	(70%)	(15%)	(9%)	(6%)	

**Child age range at baseline**		Age group 1	Age group 2		
		*n* = 41	*n* = 47		
		Age range = 15–22 months	Age range= 33–39 months		
		(M=19 months, SD= ±2.17 months)^**^	(M=36 months, SD=±1.87 months)^**^		

**Child ASQ**–**communication subscale possible delay (grey/black zone)**		Multilingual group	SLCN group		
		*n* = 6/26	*n* = 37/62		
		(23%)	(60%)		

*Note*: *87 families participated, including 88 caregivers and 88 children. One family had different caregivers for the text messages and SLT sessions. Another family with twins provided baseline information for both children but completed the acceptability questionnaires once, considering both twins.

** Both age groups are reported combined.

Abbreviations: IMD, Index of Multiple Deprivation (a neighbourhood‐level proxy for socioeconomic circumstances); ASQ, Ages and Stages Questionnaire.

### 2.3 Service Pathways

Figure 1 shows participant pathways through the study. The universal text‐message service shared a link to a BBC Tiny Happy People video each week. The videos illustrated evidence‐based ideas for supporting early child language, having been co‐developed with caregivers and professionals (www.bbc.co.uk/tiny‐happy‐people—accessible in the UK). We describe this universal preventative provision as a ‘service’ throughout. The additional, targeted intervention involved a phone/video call with an SLT who followed the ELIM‐I intervention adapted for use online (McKean et al. 2022; McKean et al. 2023a).

#### 2.3.1 SLCN Group

***Universal Component***: First, to gauge *prospective acceptability*, and ensure it was appropriate to continue, caregivers were sent three videos (Supplementary Information C) and asked how they would feel about receiving them via a text service. This prospective acceptability step was added for this group only because many caregivers reported speech and language delays in their children, with over half scoring below the communication subscale cutoff on the Ages and Stages Questionnaire. Enrolling these families in what is an age‐based text service without first consulting them could therefore have been inappropriate or distressing. Next, to determine *retrospective acceptability*, caregivers were invited to a weekly text‐service for one month. Content was selected by an SLT to be more developmentally appropriate (language‐wise) for the child than the universal service, according to the caregiver's description of their child's communication as *prelinguistic*, *one‐word*, or *multi‐word* (full content: Supplementary Information D).

***Additional Targeted Support: Online ELIM‐I***: To test the acceptability of the targeted support, we pseudo‐randomly selected half the SLCN group (ensuring representation of neighbourhood deciles, motor and communication delays) and offered them a 30‐min phone/video call with an SLT. This involved a guided conversation and language screening using a digital adaptation of the ELIM‐I measure for children over two years (McKean et al. 2022; McKean et al. 2023a) and the Language Use Inventory for children under two (O'Neill 2002). Those ineligible for further support were offered a ‘Universal pack’ of Talking2gether PCI strategies and the month's text service. Those eligible for further support had a second call introducing Talking2gether PCI strategies, with the SLT and the participant working collaboratively to select a single strategy to integrate in daily routines. A follow‐on support route (self‐directed or coached) was determined based on a Capability, Opportunity, Motivation, and Behaviour framework (Michie et al. 2011) discussion with up to four video/phone calls alongside the text service (see Supplementary Information E for information about the SLT sessions). The final calls involved troubleshooting challenges and discussing the impact of the implemented strategy. Twenty (of 28) participants accepted this offer (the ‘SLCN SLT’ group).

#### 2.3.2 Multilingual Group

To allow us to later gauge *retrospective acceptability*, caregivers received the text‐message service for 3 months (Full content: Supplementary Information F). Each month, in addition to three videos with age‐based content, one video celebrated and supported multilingualism.

### 2.4 Acceptability Questionnaires

Using online questionnaires, caregivers rated service acceptability across the seven dimensions of the Theoretical Framework of Acceptability (Affective Attitude, Burden, Ethicality, Intervention Coherence, Opportunity Costs, Perceived Effectiveness, Self‐efficacy). Questionnaires were adapted slightly for each group and timepoint. Free text options gave caregivers space to expand. Figure 1 shows completion rates. The SLCN group received a prospective acceptability questionnaire with questions about the videos and text service separately (Supplementary Information G) and a retrospective acceptability questionnaire about the service as a whole (Supplementary Information H). The SLCN SLT group also received a questionnaire about the targeted ELIM‐I intervention (Supplementary Information I). The multilingual group received a retrospective acceptability questionnaire about the service as a whole (Supplementary Information J).

### 2.5 Semi‐Structured Interviews

‘Virtual face‐to‐face interviews’ were chosen for caregiver convenience, capturing non‐verbal cues and ease of recording and transcription (Hanna and Mwale 2017). All participants were invited to an interview, which was conducted in English by a female research assistant (first author EH), with whom participants had prior contact. Each semi‐structured interview (approximately 30‐min) was video‐recorded for semi‐automated transcription via Google Meet. Topic guides were reviewed by co‐authors and adapted slightly for each group and timepoint.

***SLCN Group***: To explore prospective acceptability, those who had not been pseudo‐randomly selected for SLT sessions (*n* = 29) were invited to online interviews (Topic guide: Supplementary Information K). Caregivers shared their thoughts about the videos, the text‐message service and potential improvements, particularly regarding SLCN and multilingual content (where applicable). Twelve of 29 caregivers participated. This group was also invited to retrospective interviews (Topic guide: Supplementary Information L). Six of 12 caregivers participated in these. Participants who took up the SLT offer were invited to retrospective interviews to explore the text service and the SLT sessions (Topic guide: Supplementary Information M). Ten of 17 caregivers participated.

***Multilingual Group***: To explore retrospective acceptability, all caregivers were invited to interviews that explored the text service and video content with a focus on multilingualism (Topic guide: Supplementary Information N). Eleven of 26 caregivers participated.

### 2.6 Data Analysis

Table 2 provides descriptive statistics summarising the acceptability questionnaire responses. Reflexive thematic analysis was conducted on interview transcripts (Braun and Clarke 2006; 2019). This suited our goal of interpreting families' attitudes and experiences and identifying patterns with the timeframe available (Braun and Clarke 2021). After rewatching recordings and correcting automated transcripts, the data were coded and codes grouped to develop initial themes. Themes were continually refined by revisiting transcripts, recordings, and member‐checking. Primarily, we used an inductive approach with both semantic and latent coding. However, a deductive element linked identified themes to the research questions (Byrne 2022).

We recognise that themes reflect researcher interpretation (Braun and Clarke 2019). We adopted a constructivist perspective determined by the emphasis caregivers placed on their contributions and researcher judgement during analysis (Byrne 2022). An experiential orientation was appropriate to capture families' attitudes and opinions regarding their experience (Byrne 2022). The primary researcher (EH), who conducted all interviews and the SLCN group's thematic analysis, is white, middle‐class, university‐educated, and has no personal experience of parenting. The same applies to RMI, who conducted the multilingual thematic analysis. EH had prior experience running acceptability interviews.

During analysis, we used a collaborative approach, employing member checking and peer debriefing for a richer data representation (Braun and Clarke 2019; Byrne 2022). For member checking, all interviewed caregivers received a summary of their group's themes. Three caregivers responded and all approved the summary. For peer debriefing with the SLCN group, DM reviewed 10 interviews (*prospective acceptability*: 4 random and 1 of a participant who found the service unacceptable; *retrospective acceptability*: 5 random). For the multilingual group, EH reviewed 4 randomly selected interviews.

## 3 Results

### 3.1 Quantitative Analysis: Acceptability Questionnaires

As shown in Table 2, average acceptability was high on all seven dimensions of the Theoretical Framework of Acceptability for all pathways. Nonetheless, the service was unacceptable for one caregiver in the SLCN group, a finding discussed below. Acceptability was particularly high for the targeted ELIM‐I intervention, demonstrating caregivers valued this online support. For the multilingual group, acceptability was also high albeit with slightly lower ratings for *opportunity cost* and *burden*, which relate to the time/effort of engaging.

**TABLE 2:** Prospective and retrospective acceptability of the service for families with SLCN and multilingual families.

	General acceptability	Affective attitude	Burden	Ethicality	Intervention coherence	Opportunity costs	Perceived effectiveness	Self‐efficacy
*Prospective acceptability of the videos: SLCN group (N = 56)*								
Median	4.50	4.33	4.50	4.00	4.33	4.40	4.50	4.50
(Min–max)	(1–5)	(3–5)	(3–5)	(2–5)	(3–5)	(2–5)	(3–5)	(3–5)
% Favourable ^*^	86.2	91.4	94.8	89.7	89.7	94.8	91.4	96.6
*Prospective acceptability of the text service: SLCN group (N = 56)*								
Median	5.00	4.50	4.50	5.00	5.00	4.17	4.50	4.00
(Min–max)	(3–5)	(3–5)	(3–5)	(3–5)	(2–5)	(3–5)	(3–5)	(3–5)
% Favourable^*^	96.6	96.6	96.6	94.8	86.2	89.7	87.9	91.4
*Retrospective acceptability of the text service and videos: SLCN group (N = 25)*								
Median	5.00	4.54	4.60	5.00	4.67	4.40	4.33	5.00
(Min–max)	(3–5)	(4–5)	(4–5)	(4–5)	(3–5)	(4–5)	(3–5)	(4–5)
% Favourable^*^	96	100	100	100	96	100	92	100
*Retrospective acceptability of targeted ELIM‐I intervention: SLCN group (N = 16)*								
Median	5.00	5.00	4.50	5.00	5.00	4.40	4.75	5.00
(Min–max)	(4–5)	(4–5)	(4–5)	(3–5)	(3–5)	(4–5)	(3–5)	(3–5)
% Favourable^*^	100	100	100	93.8	87.5	100	93.8	93.8
*Retrospective acceptability: Multilingual group (N = 25)*								
Median	4.40	4.38	4.20	4.00	4.33	4.00	4.00	4.00
(Min–max)	(3–5)	(3–5)	(3–5)	(2–5)	(4–5)	(2–5)	(3–5)	(4–5)
% Favourable^*^	96	96	84	96	100	76	88	100

^*^Score above 3 on scale from 1–5.

### 3.2 Qualitative Analysis

Since interviews with caregivers in both groups revealed many common themes, the following analysis consolidates findings, highlighting differences where necessary. Three overarching themes were identified (Thematic map: Supplementary Information O):

1. *Demand for trustworthy guidance to address uncertainty* revealed an appetite for a service that could address caregivers' concerns and curiosity.
2. *Positives of the support service* identified what caregivers valued.
3. *Catering for diversity* identified adaptations required to adequately serve diverse families.

Where quotes are provided, participant number indicates group membership (M = multilingual group, S = SLCN group, including multilingual families with SLCN).

#### 3.2.1 Demand for Trustworthy Guidance to Address Uncertainty

Caregivers reported that child language development was important to them but expressed uncertainty around supporting it (whether experienced as curiosity or anxiety). Some expressed concern regarding SLCN. One stated that *“it's hard to know what will come on its own developmentally—they'll just get there with it and it's just because of his age—or whether it's something where there is a gap where we need to be dealing with that now while he's younger rather than waiting till he's older”* (S48). Others knew there was an identified SLCN and the difference in their child's developmental trajectory caused them to *“worry about the future”* (S31). In the multilingual group, despite feeling it was *“extremely important”* (M96) to raise their children hearing home languages, caregivers were concerned *“to not overwhelm them”* (M8) and wondered how to navigate this. They appreciated guidance as *“when I try to read something about it, there's not that much out there”* (M8).

Many caregivers felt the digital service could provide the trusted, UK‐based guidance they sought in a non‐judgemental way. One noted that, previously, they hadn't *“really found anything that's professional and structured”* and that *“It's this proper information that's come from proper sources and it's tried and tested*” (S31). Another noted “*the problem I find with googling is it comes back quite negative and then you end up on a lot of American sites and stuff … And I'm always trying to find kind of NHS sites or things that are relevant to living in this country”* (S48). Evidence‐based guidance was emphasised as particularly useful for caregivers with young children, for first‐time caregivers and those with concerns regarding SLCN but not currently eligible for support or on waiting lists.

Caregivers felt receiving the service earlier would allow for a longer period of implementation and would help during times of less experience and greater uncertainty, when caregivers are more likely to seek guidance. As one explained, *“if we'd been having them since he was very young then I can see how we would have really applied a lot of them and that would have been really good”* (S73). One caregiver suggested *“an opt‐in service from the get go. Your baby's been born, you generally see your health visitor within the first week. Opt in from that week”* (S51). Another first‐time caregiver explained as a first‐time parent, *“I don't have that experience. Rather than having a book that I feel is just too much information or kind of a website that I have to go and hunt out, I'm actually just kind of having something delivered to me every week going, ‘Have you thought about this? Here's a new idea’”* (S26).

Of those waiting for speech, language and communication support, one caregiver stated they had *“waited nearly 18 months between his last appointment, so to get something weekly is actually really beneficial”* (S43). This provides caregivers with *“next steps”* and *“a goal now”* (S91).

Overall, families wanted the service from early on. Caregiver uncertainty regarding language development—especially the unique aspects of SLCN and multilingualism—were key drivers, with value placed on access to non‐judgemental, evidence‐based and UK relevant information.

#### 3.2.2 Positives of this Support Service

Positives identified by caregivers fell into three sub‐themes: *suitable for busy family life*; *beneficial, practical, reassuring and enjoyable; and value of personalisation and human connection*.

**Suitable for Busy Family Life**: The text messages offered flexibility in accessing and sharing content. One caregiver felt it was “*quite handy cause they're there for when you can actually watch them cause like the phone calls we get through other services, sometimes you're not always available. But the text message you can just click back when you are available or you've got like a couple of minutes*.” (S43). This was valued given busy or unpredictable family routines. Easy sharing of content was valued as “*all the family members involved can then go, ‘Oh that's what they're doing. We can do it too’*” (S43). This reflects the reality of shared childcare. The flexibility of online SLT sessions was also valued because “*if I was at the park and [childname] was running around the park, I could still take the call*” (S51) and meant they “*didn't have to worry about a stranger coming to my house*” (S51).

Short video content was emphasised as essential to caregiver engagement. Having one video per week made information easier to consume, digest and implement. This caregiver noted that they would *“make that time each week to be like we're going to watch this and let's try … a few activities”* (S61). They appreciated that each video focused on *“a single idea, which when your brain is full of a million things is like just give me one thing rather than six and then I'm going, ‘Uh there was something really useful and I can't remember any of it’”* (S26). Although some caregivers appreciated the option of additional materials such as articles linked on the BBC website, for most, videos were preferred as *“the article itself is quite long”* (S51). Nonetheless, transcripts and captions were often valued. One caregiver found that when they were not able *“to watch the videos … being able to read the transcripts kind of meant I got all the information I needed”* (S26), while others found it aided comprehension to both listen and read “*because that's how I tend to listen best”* (S94).

The majority of caregivers noted it was important the service remain free and their only barrier to engagement would be *“if there was a cost attached to it”* (S58). Similarly, it was crucial that videos featured easily accessible everyday items and places for implementing ideas. Caregivers viewed this positively, noting that some tips required *“literally just your own voice”* or *“just things you had in the house”* (S43).

**Beneficial, Practical, Reassuring and Enjoyable**: Caregivers frequently commented on how the service benefited them practically and emotionally, attributing improvements in family communication to it. One noted that *“[childname]’s speech is coming on. The proof is in the pudding”* (S51), whilst another explained how the service *“helped me expand my language or the way I'm speaking to him”* (S58). There was a feeling this built confidence and reduced problematic episodes: *“kind of helped us to know how to react to him… he gets a lot less frustrated now than he used to”* (S39).

Caregivers appreciated the focus on practical guidance, rather than on diagnosis: “*what I liked about the videos actually as well is that rather than a focus on what the issues are it's like providing guidance on tips to try and help build their confidence and your confidence with them*” (S73). Activity ideas were popular to “*vary it up a little bit*” (S58) and bring “*quite a bit of joy*”, with some noting that they were “*just having a bit more fun*” now (S33). The service illustrated opportunities for connection and the possibility of enjoyable everyday activities. One caregiver noted how watching the videos made them feel “*nice to think about connecting with your child and seeing people doing the things that you could, then think ‘oh I'll do that with mine’*” (S64). Another realised the value of involving their child in everyday activities like cooking and cleaning, noting how, previously, the “*screen … was my go to, if I need to clean, if I need to cook*” (M74). After engaging with the service, they realised they “*can actually involve him and he was really happy with it, which was surprising*” (M74). This highlights how the service helped families to support language with practical tips in the context of enjoyable everyday life.

Another benefit was providing the reassurance caregivers sought. For multilingual families, the videos helped caregivers realise their child's brain *“will manage to adapt”* (M74) and for one caregiver *“this gave me confidence to continue doing what I was doing”* (M96). Examples of early communication helped caregivers notice what their child could do already such as *“learn parts of the face”* (S58). This reassurance was particularly notable for the SLT sessions, regardless of the number of sessions families had. One caregiver noted, *“it's very reassuring to hear from somebody in that profession from what they have heard about your child, they don't think there's any reason for concern”* (S23). Another felt that if they'd “*had something like this to refer to I think it would have raised my awareness a bit more or helped me sort of identify that oh yeah, something's just not quite right here.”* (S58). Similarly, the targeted ELIM‐I *“helped put in a referral for [childname]”* (S63) who needed additional support.

The SLT sessions offered other benefits in terms of prioritisation and personalisation. For example, one caregiver felt that while the videos *“just solidified … all stuff that we're kind of doing already”*, they *“got some new bits from talking with [SLTname]”* such as *“giving time to answer”* for their child (S63). Another noted how they were more relevant for a child with complex needs than generic resources which are *“difficult because she's got such a wide range of developmental strengths and delays that there's always something that's relevant and something that isn't quite mixed together”* (S63). Overall, several caregivers emphasised how they valued the SLT clarifying things and tailoring suggestions. They felt these benefits regardless of other support they received outside the study. For example, selected videos made this caregiver feel *“hopeful, more than anything because … he's under a speech and language therapist as well and sometimes where it's a half an hour session of just ‘say this say this say this’ he gets very very grumpy”* whereas *“he's a bit more engaging”* with this service (S39).

**Value of Personalisation and Human Connection**: The service's personalised, friendly and human qualities were valued by caregivers. Using first names in text messages fostered a sense of support, making them feel *“like you're getting messages from a friend”* (S62). One caregiver from the multilingual group shared that *“we normally don't have family and relatives where we live and it's really hard sometimes. So, when you receive text messages like this, using your child's name, it just comes close to the heart, really makes the difference, than just a random text”* (M96). Referring to children by name also helped caregivers with multiple children identify which child a message related to, meaning *“you know which kid it's for”* (S43). Caregivers who received the targeted ELIM‐I particularly appreciated *“somebody to talk to that was specifically tailored to her speech but was non‐judgmental.”* (S51). They described the sessions as feeling informal and friendly which *“just makes you feel so at ease”* (S46).

Caregivers had a strong preference for a human presence behind the service. One stated, “*it was really nice to know that there was actually a person behind the text messages because if you just get text messages that do have all the same format, it can… feel a bit robotic*” (S39). This human element provided a sense that “*someone was listening… that there was someone at the end of it that I could reach out to if I needed help” and being viewed as “more of a help service than a text service*” (S39).

Overall, caregivers observed a wide range of benefits, some specific to digital delivery. Nonetheless, the next theme makes clear that there are further improvements that would be beneficial or necessary for acceptability.

#### 3.2.3 Catering for Diversity

The final overarching theme revolved around family diversity. Sub‐themes were: *importance of being represented, consequences of lack of representation and necessary changes*.

**Importance of Being Represented**: Caregivers appreciated the inclusive video content that was relatable because it featured diverse family structures, accents, and cultures. They particularly enjoyed content that featured families directly rather than experts talking to camera. One caregiver explained that they: *“quite like as well you know different accents, different types of people, different family setups.”* (S64). Most useful for many was seeing videos relating to their own family situation. Seeing other multilingual families made one caregiver feel *“connected with the people”* and *“touched”* by the videos like *“this is really talking to me because it's what we live everyday as a multilingual family”* (M96). Likewise, one caregiver in the SLCN group, explained that SEN specific videos would *“be wonderful because then … the level of expectation would be different”* and that *“if there was a SEN section that I could then kind of finish at, I might have then been brought back down to my norm and been like, okay, these are our people”* which would be *“extremely useful and more tailored to us than a generic speech‐language video”* (S31). Thus, there is scope for further diversity of content.

**Consequences of Lack of Representation**: Of those who wanted content to be more diverse, one felt strongly that “*they miss the point that some people are neurodiverse, some people have other disabilities, such as myself, with being deaf. I think they don't understand that every child is unique, they're just grouping them … And I think the BBC, while their intentions are good, they fail to recognise that everybody is not a one‐size‐fits‐all*.” (S81).

Lack of enough inclusive content diminished caregivers’ ability to apply tips and affected them emotionally. One noted the need for more inclusion of siblings, as the tips were *“harder to implement in real life unless you've got that kind of full‐on one‐on‐one time”* which is *“impossible with another child”* (S61). Another suggested showing multiple scenarios for applying tips to avoid the burden on caregivers to *“be creative with that”*, noting a video that mentioned *“take your child to the park to talk to the ducks. It's like we can't do that here”* and so it would be helpful to say, *“you could do this, this or this which would have the same developmental support if you don't have that particular context”* (S26). This illustrates a sub‐theme of the burden of translating abstract advice into practical implementation and how relatable content can mitigate that.

Having felt their situation was unrepresented, some caregivers in the SLCN group found current videos unhelpful and distressing, leading to feelings of inadequacy. This emotional reaction occurred both when viewing content aimed to be more developmentally appropriate for their child's language stage (albeit featuring younger children) and content that matched their child's age (but was too advanced language‐wise). One caregiver felt that *“these videos actually make you feel like a shitty parent, that you're failing because your child is not meeting them milestones*” (S81), leading them to withdraw from the study halfway through the month‐long text service. This highlights the critical need to increase representation of diverse development and parenting circumstances in video content and address the risk of unintended negative consequences for families.

Overall, feeling represented was important not only to make the service more effective but importantly to avoid harm.

**Necessary Changes**: Caregivers acknowledged the challenge of creating a universal service, recognising *“it's hard cause even SEN is such a range itself”* meaning *“there's no pleasing everybody.”* (S31). However, several possible improvements to the current service were identified.

There was a strong sentiment regarding the need to create content that is for both the age *and* developmental stage of children with SLCN. One caregiver explained their experience of watching content that matched their child's language stage but featured children far younger than theirs: *“Seeing it with a baby, if you're working with a baby, is great, but seeing it with a toddler, you are just sat there going, ‘But she's not a baby.’”* and that *“if you can then see that actually this works with a child that does have these developmental delays… that would be more helpful”* (S51). Another suggested that by showing multiple children of different ages but at the same given language stage *“people can see that actually it can be used for all ages”* (S43). Content adapted to the reality of SLCN would help to avoid distress and make tips easier to implement.

Given the current relatively limited availability of SLCN‐adapted content, we asked caregivers whether they would rather receive chronological‐age or language‐stage based content and found that responses varied according to complexity of need and personal preferences. For families whose children had less marked SLCN, age‐based videos tended to be preferred. This caregiver explained they *“like the idea that they are personalised … for his age”* (S14). For those with more complex SLCN, families tended to choose either a stage‐based approach or a mixture of age‐ and stage‐based videos. One caregiver explained that their family needed *“100% a more personalised approach simply because [childname] obviously isn't a typical three‐year‐old… I would worry that the videos I was receiving, he would be nowhere near that level yet”* (S31). Whereas another caregiver preferred a hybrid approach, stating *“it'd be nice to get ones that were appropriate for where he is now and if he is a little bit behind, you know to see those ones, but also to see where you kind of should be and that might be quite reassuring that he's not as far as behind as you sometimes worry like sometimes wonder”* (S48). To better tailor content, caregivers suggested leaving open “*the option for the parent so the parents can choose what's appropriate for their child instead of automatically having it in depending on the age”* (S14). Another suggested having a short questionnaire at sign‐up that *“directs you to like whichever bit would be appropriate for you”* (S58). Multiple caregivers also suggested regular check‐in texts to adjust the content level. For example, *“being able to kind of … message back and saying, in this category, can we jump ahead… or jump backwards”* (S26). Until more age‐and‐stage appropriate videos are available, caregivers suggested improvements like removing ages from videos or organising content by developmental stages like *“a first word user or combiner”* to avoid causing distress (S91). However, it was clear that these are only partial solutions and there is a need for more content that demonstrates support strategies for children with SLCN of various types. Finally, most multilingual families found the age‐based content appropriate for their child, with feedback like *“the selection was spot on”* (M96) and *“well timed”* (M54), although some noted developmental levels vary across languages. One observed their child was more advanced *“in the English part”* (M6). Therefore, support for adapting to children's level in each language might be valued.

The second type of change involved increasing accessibility with translations and adaptations for caregiver neurodiversity. Although most multilingual families in this study (who were all proficient in English) preferred content in English, one explained that *“when I moved here, I struggled to understand things properly”* (M54), noting translations would help where English is a barrier. Some families wanted their languages represented: *“It would have been great to see how that plays out with people similar to myself”* (M6). Others wanted examples that go beyond simple bilingualism, where a *“household speaks multiple languages”*, with each parent speaking a different non‐English language or dialect (M3). Content for navigating tonal languages was also requested since *“accents and tonations would make certain words sound different.”* (M6).

In terms of caregiver neurodiversity, one participant mentioned their partner's limited scope for engaging with and implementing ideas due to ADHD and Autism, noting their difficulty with responding to their child's babble because *“their brain doesn't pick up on it”* (S51). Alongside comments from a deaf parent this highlights the need to accommodate and represent the diversity which exists amongst caregivers.

To summarise, representative content was crucial for the service to be of practical use and to avoid causing distress. Additional content catering for SLCN, as well as more representation of multilingual families and caregivers with Special Educational Needs and Disabilities (SEND), would likely foster implementation.

## 4 Discussion

This study explored the acceptability of a universal digital service that shared BBC Tiny Happy People video content with families via a weekly text message and, where appropriate, supplemented this with a targeted, online ELIM‐I intervention. We sought input from families with children who have SLCN and/or do not speak English primarily at home. Average quantitative ratings were high for all seven dimensions of acceptability and qualitative analysis suggested a real appetite for the service. However, critical additions to content were noted, especially videos featuring multilingual families and examples of supporting children with SLCN.

Caregivers generally felt the service met their needs, addressing the curiosity and concern they felt about child language development and their prior difficulty in finding relevant support online or in‐person. They commented on their trust in the BBC content in a UK context. A caveat here is that, while a service like this was said to allay concerns for caregivers whose children had mild SLCN, for some caregivers whose children had more complex SLCN the contrast to evoked norms was striking and uncomfortable. Caregivers suggested solutions that should be explored further. These generally involved creating materials that would make it easier to see how to enter into the Zone of Proximal Development of a child with SLCN so as to use language in a developmentally‐attuned way (Tamis‐LeMonda et al. 2014).

Some caregivers implied they interpreted the advice as meaning there was ‘proof’ of there being a ‘proper’ way to do things. This suggests a risk that the service could amplify or create narrow child development and parenting norms and fuel anxiety or a feeling of being judged (Nitsche et al. 2021; Ipsos MORI 2020). It is incumbent on those designing and delivering any service to strive to communicate the extent, meaning and limits of evidence‐based recommendations, and how these oughts to complement varied parenting approaches.

Many positive features of the service were identified. Uniquely, the concise and clear content delivered via text message made it convenient for busy families. The value put on this, and on the reputable source, is consistent with the findings of an Australian evaluation of a social‐media service sharing similar child language support messages (Dunstan et al. 2024). Another key strength was the human connection. For a sensitive topic like child development, personalised texts responded to by a real human boosted feelings of support and engagement. For some families, the SLT sessions provided even greater perceived benefits, enhancing caregivers' personal awareness, clarifying messages and tailoring advice for children with diverse needs. This suggests that SLT sessions are an important complement to the text service and supports the approach of stacking interventions in a preventative public health framework (McKean and Reilly 2023b). The current pathways allowed for quick triaging and might be used to take pressure off existing services and support carers on waiting lists (although economic evaluation would be needed). Importantly, perceived benefits persisted regardless of whether caregivers were accessing other support, indicating the service can work alongside existing systems.

Caregivers felt the service positively impacted their interactions with their children, offering ideas for ways to connect that could be easily imitated. This is in line with Pila et al. (2019) who explored text messaging services with American parents and argued the use of video models would promote observational learning and behaviour change (at least to the extent that the model is attended to, retained, felt to be do‐able and perceived as desirable, given Social Cognitive Theory: Bandura 2001).

Some caregivers noted that the ideas were not limited to traditional games and outings but also involved including children in everyday routines like cooking. That caregivers noted how novel this latter approach felt highlights a common Western habit of confining learning to purpose‐made activities such as puzzles designed to teach animal names (Rogoff and Mejia‐Arauz 2022). The service instead often promoted learning through shared purpose in everyday household tasks, a powerful method used across many cultures that provides ideal conditions for child language learning (Tamis‐Lemonda and Masek 2023). Caregivers reported they had overlooked this approach but were keen to adopt it. Its high acceptability may be because it builds in personalisation by encouraging families to embed advice within existing routines. Learning through everyday activities is likely to be more universally applicable and feasible than purpose‐made activities given social disadvantage negatively impacts the amount of time caregivers spend interacting one‐to‐one with their child (Monna and Gauthier 2008).

The strongest sentiment regarding recommended changes was the need to diversify content. This came up despite significant co‐development and consideration of diverse family preferences from the outset, highlighting the need for an iterative approach to service development with continued parent and caregiver input. Creating resources that include varied developmental trajectories, SEND, SLCN, and multilingualism would make it easier for caregivers to implement suggestions and prevent emotional discomfort. While caregiver choice over content can help, age‐and‐stage‐appropriate videos are needed for this choice to be meaningful for families whose children have SLCN. These videos could feature multiple ages at the same developmental stage and vice versa.

## 5 Strengths and Limitations

This relatively large acceptability study represented a wide array of developmental conditions and home languages with families from the four nations of the UK, living in diverse socioeconomic circumstances and family structures. Nonetheless, it has limitations in that all participating caregivers were parents, including only two fathers, and no foster parents, which limited the range of caregiving perspectives captured and may have biased findings towards maternal experiences and priorities. All caregivers who provided feedback were comfortable using English as no translations were available; this likely resulted in the under‐representation of families who experience language barriers with English‐based services, for whom acceptability may differ systematically. Future research could explore acceptability when the service is delivered in other languages, such as Welsh, British Sign Language and languages that would make the service accessible to caregivers with limited English‐language skills. Furthermore, acceptability could also be explored more thoroughly in a group representing caregivers who are neurodivergent and/or have lived experience of physical, mental or learning differences that could affect their experience of the service. Finally, because participation was voluntary, the findings reflect the views of families with the time and motivation to engage in research and may not be representative of all families using the service.

## 6 Conclusion

This mixed‐methods study found significant demand for a digital early years language support service among families whose children had SLCN and/or were multilingual. The service, combining universal text messages with targeted intervention for those with or at risk of SLCN, was generally highly acceptable, suggesting potential to complement in‐person services. Targeted SLT support was highly valued. However, video content should be expanded so families can identify with examples and implement strategies. More content that is both age‐ and stage‐appropriate for children with SLCN would increase applicability and help avoid caregiver distress. Resources supporting and celebrating multilingual households were valued by families speaking languages other than English at home, and there is scope to expand them further by featuring more than two home languages, tonal languages, and children more advanced in one of their languages.

## Funding

The study was funded by a University of Sheffield Knowledge Exchange grant and ethical approval was received from the School of Psychology Ethics Sub‐committee at the University of Sheffield in October 2024 (058667).

## Conflicts of Interest

The authors declare no conflicts of interest.

## Data Availability

The data that supports the findings of this study are available from the corresponding author upon reasonable request, and supplementary materials are available at OSF: osf.io/ty9d7.
